# Modeling the Commuting Travel Activities within Historic Districts in Chinese Cities

**DOI:** 10.1155/2014/253289

**Published:** 2014-11-04

**Authors:** Mao Ye, Miao Yu, Zhibin Li, Fengjun Yin, Qizhou Hu

**Affiliations:** ^1^Department of Transportation Engineering, Nanjing University of Science and Technology, 200 Xiaolingwei, Nanjing 210094, China; ^2^School of Transportation, Southeast University, 2 Sipailou, Nanjing 210096, China

## Abstract

The primary objective of this study is to analyze the characteristics of commuting activities within the historical districts in cities of China. The impacts of various explanatory variables on commuters' travels are evaluated using the structural equation modeling (SEM) approach. The household survey was conducted in the historical districts in Yangzhou, China. Based on the data, various individual and household attributes were considered exogenous variables, while the subsistence activity characteristics, travel times, numbers of three typical home-based trip chains, trip chains, and travel mode were considered as the endogenous variables. Commuters in our study were classified into two main groups according to their working location, which were the commuters in the historic district and those out of the district. The modeling results show that several individual and household attributes of commuters in historic district have significant impacts on the characteristics of travel activities. Additionally, the characteristics of travel activities within the two groups are quite different, and the contributing factors related to commuting travels are different as well.

## 1. Introduction

Historic district has many familiar names in China such as old town and ancient city. The Norms on Protection of Historic and Cultural City Planning were issued in 2005 which defines that the historic area should reflect a historic development process or a range of development. In recent years, with the rapid development of urbanization and motorization, traffic demand has been increased dramatically. Under such background, traffic problems in historic district are increasingly severe. How to coordinate the relationships between city protection and traffic development becomes an important topic since it is very important for the sustainable development.

Because of the special protection towards the historic and cultural heritage, mass basis construction in transportation infrastructures is usually not allowed. Therefore, the supply of transportation is very limited to meet travelers' needs, and the traffic congestions frequently occurred in the historic center, especially in the morning and evening peaking hour. As a consequence, the analysis on travel characteristics of the region's residents, especially commuters, is important for the alleviation of the traffic jams. Besides, it is particularly meaningful for policy makers to develop effective traffic strategies. The commute trip, which is known as a spatial movement from home to working place, often accounts for a great proportion in commuters' daily trips (nearly 50%). Thus, the solution of commuters' travel problem would be very helpful for the soothing of traffic congestions on roads.

There are a considerable number of studies on the characteristics and influencing factors of commuting travel activities, but most cities they researched do not have historic sites. In China, the historic district usually has a high population, a mixed land use pattern, and a different density of road network, and all these are quite different from those of the entire city. Besides, these factors are confirmed to be of particular importance to characteristics of commuters travel. Therefore, it is very necessary to investigate the relationships between commuters' characteristics, activities, and travel behavior.

Activity-based approach on travel behavior usually focuses on activity and decision-making, analyzing, and modeling relationships between travel behavior and activity [1–6]. In order to elaborate on the study of travel activity patens and influencing factors, these activities should be categorized at first. Some scholars suggested that they could be divided into three parts based on travel purpose: subsistence activity, involuntary activity, and voluntary activity [7]. Meanwhile, further studies proposed a more efficient classification approach, which distinguished them into four categories: subsistence activity, maintenance activity, discretionary activity, and others [8–11]. This research adopted the latter one. Moreover, considering the activity characteristic of commuters, we distinguished them into subsistence activity and nonsubsistence activity.

According to previous studies, individual and household decision-making are dominant influencing factors on commuters travel behavior [12–17]. An insight on the mechanism of commuters travel behavior, individual and household, is very meaningful for scholars to understand commuters' travel. A lot of research has been done on the issue, and many models have been proposed (such as calculation process model, the discrete choice model, etc.). But few models can truly explain the complex relationships among them. Structural equation modeling (SEM) is a popular statistic approach in 1960s. It can test and estimate causal relations with a combination of causal assumptions. Unlike the traditional models, SEM can model two types of variables: observed variables that are directly collected or measured and latent variables that are not directly observed or measured. For its flexibility, foreign scholars have been introducing the SEM to analyze the complex travel behavior [18–22].

In this study data were obtained from the household survey in the historic district of Yangzhou. A structural equation model (SEM) was developed to explore the relationships among commute trip-activity characteristics, travel behavior, and individual and household attributes. A classification was done according to commuters' working location, which is convenient for a further comparison of their respective influencing factors. The remainder of this paper is organized as follows.  Section 2 presents the data source used in the research and exogenous and endogenous variables.  Section 3 is mainly about descriptive statistics of the data, using the statistical analysis methods to discuss travel characteristics of those two groups.  Section 4 presents the methodology of structural equation model and the modeling framework. Then, in Section 5, we discuss the model estimation results, and in the final section, conclusions are summarized and discussed.

## 2. Methodology

The general SEM assumes that causal relationships exist among a set of latent variables, which are specified as linear combinations of manifest variables. Through the validation of the covariance among the manifest variables, the coefficients of linear regression model can be estimated to confirm whether the assumed model is suitable for analysis. If the result is fit, the assumed relationships among the latent variables are reasonable. There are some steps involved in SEM construction. They are as follows: establish the conceptual model, compose a path diagram, specify the variables, select the input matrix model, evaluate the sample size and its effects, and identify the methods for model (such as the approach for estimation, evaluation, and modification), as well as cross-validity.

The SEM is composed of measurement equations and structural equations. Theoretically, a standard SEM has three equations and it could be expressed as below:

(1) $$\begin{array}{c} \eta =Β\eta +\Gamma \xi +\zeta , \end{array}$$

(2) $$\begin{array}{c} y=\Lambda_{y}\eta +\varepsilon , \end{array}$$

(3) $$\begin{array}{c} x=\Lambda_{x}+\delta , \end{array}$$

where*η* is vector of latent endogenous variables;* Β* is the coefficient matrix of direct effects between endogenous latent variables; Γ is the matrix of regression effects for exogenous latent variables to endogenous latent variables; *ξ* is vector of latent exogenous variables; *ζ* is error vector of structural equation; *y* is vector of observed endogenous variables; Λ_*y*_ is the matrix of structural coefficients for latent endogenous variables to their observed indicator variables; *ε* is vector of measurement error terms for observed variables *y*; *x* is vector of observed exogenous variables; Λ_*x*_ is the matrix of structural coefficients for latent exogenous variables to their observed indicator variables; *δ* is vector of measurement error terms for observed variables *x*.

Equation ((1)) is the structural model of exogenous and endogenous variables, ((2)) is measurement model of endogenous variables, and ((3)) is measurement model of exogenous variables. Actually, the objective of employing the SEM in the study is to estimate the whole set of coefficients contained in the above 8 matrixes, which could be set as a fixed one or a free one.

A complete SEM consists of 8 coefficient matrixes: Λ_*y*_, Λ_*x*_, *Β*, Γ, Φ, Ψ, Θ_*ε*_, and Θ_*δ*_. Γ is the covariance matrix of latent variable *ξ*, Ψ is the covariance matrix of error term *ζ*, Θ_*ε*_ and Θ_*δ*_ are covariance matrixes of *ε* and *δ*. If the assumptions we made are held true, the population covariance matrix will be equal to the sample covariance matrix. Thus, both of the variance and covariance of observed variables (i.e., the indexes of the endogenous variables and exogenous variables) are the parameter functions of the model. Several methods can be used for estimation in SEM. The most common methods for estimation are generalized least square (GLS) and maximum likelihood (ML).

The evaluation of SEM is to examine if the model is a good fit to the data. The constantly used measure is the Chi-Square test, the value of which is calculated by fitting function. As the value of the Chi-Square test is always changing with the sample size, some researchers recommend using several indices to gauge the fitness of the model. There are two types: the incremental fit index (like GFI, AGFI, etc.) and the badness of fit index (like RMR, RMSEA, etc.). All of them can be used to validate the data and sample size. Also they can help to select suitable criteria for assumptions.

## 3. Survey Investigation

The historic district, as a part of the city, whose functional properties and local socioeconomic attributes are quite different from those of the regular city, has its own particular travel characteristics. So the study of the commuters' travel characteristics in historic areas cannot adopt the same method as that of the whole city. Research should be carried out, respectively, towards different categories of commuters. In the study, all commuters were classified into two main groups according to their working location, commuters in historic district and commuters out of the district. Therefore, the collection of data was done separately to investigate the differences of their travel characteristics.

Data used for this analysis comes from household travel survey in historic district of Yangzhou (2010). The survey was conducted in the form of questionnaire, and we distributed them in every region randomly on weekdays. The content of the questionnaires consists of two main parts: (1) individual and household characteristics, such as gender, age, occupation, annual household income, and household size and (2) travel information of all trips in a whole day, including the time taken in each trip, duration of commute time, number of trips, and travel mode choice. A total of 2000 questionnaires were delivered during the entire survey, and 1525 were returned, of which 1221 questionnaires were valid. After the selection, finally we obtained 950 valid questionnaires about commuters' travel, of which 705 were about commuters in the area and 245 were outside.

## 4. Data Description

Exogenous variables selected in the paper include commuters individual attributes (such as gender, occupation, and age) and household attributes (such as household size, number of preschool children, ownership of automobiles, and annual household income). Detailed information about those variables is shown in Table 1.

The selected endogenous variables are mainly concerned with commuters' subsistence activity and travel characteristics. The subsistence activity (mainly work trips or work-related trips) is featured by commute time, commute trip number, and duration of the commuting, while travel characteristics include the total number of trips in a whole day, numbers of three typical home-based trip chains, trip chain, and mode choice. Noticeably, a trip chain is defined as a sequence of trips that starts and ends at the household location in a whole day.

**Figure 1:**
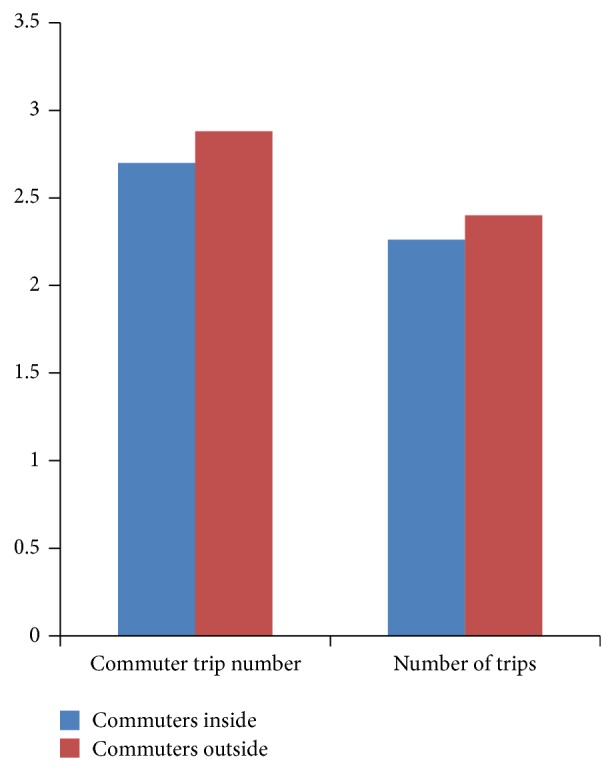
Statistical number chart of commute trips and total trips.

Figure 1 depicts the commute trip number and total trip number of commuters in historic district. In commuters' daily activities of Yangzhou city, subsistence trips take a high rate of the total trips, of which the percentage among commuters inside the district is 93.8% and the percentage among commuters outside the district is 94.2%. It reports that the nonsubsistence trips take a small proportion, so in the following classification of trip chains we only take account of the commute trips.

Finally, three major types of trip chains were used for analysis. The description of trip chains is shown as follows, where “H” denotes home, “W” denotes a subsistence (work or work-related) activity, and “O” refers to a nonsubsistence activity:

1. HWH: there is one subsistence activity within a day. Only a simple subsistence activity stop is contained in the chain,
2. HWHWH: there are two subsistence activities within a day. Commute trips with a midtrip that returns home are included, and there are no nonsubsistence activity stops,
3. HWOH: there are two types of activities within a day. Two nonsubsistence trips with a midtrip that returns home are included, and there is at least one nonsubsistence activity stop.

Table 2 shows the endogenous variables and their descriptions.

According to the statistics, there are 705 cases about commuters in historic district and 245 cases about commuters out of the district.  Table 3 shows the sample size and percentage of each class.

Great differences exist in most influencing factors of these two groups (such as travel time taken, nature of work, and travel distance), which results in great differences in their travel behaviors. Employing statistics analysis approach, we calculated the average values of commute time, duration of the commuting, commute trip number, number of trips, and number of trip chains of the two groups and took the ratios of trip chains “HWH”, “HWHWH,” and “HWOH,” respectively, to all chains. Also, Table 4 shows the ratios of nonmotorized mode, public transportation, and automobile to all modes.

According to Table 4, three obvious differences are found in the comparison of travel characteristics of the two groups. They are as follows: (1) the mean commuting duration of the former group is slightly shorter than the latter group, and the former group of commuters travels less often than the latter one. But, in the respect of the commute trip number, commuters in the historic district frequently travel for work. (2) Commuters in the district travel 2.88 times per day, while commuters out of the district travel 2.4 times per day. Similarly, the home-based chains of the former are more than that of the latter one. Observing (1) and (2), we can get the explanation that the commute distance of the inside commuters is shorter, so they have more free time and are more likely to travel. (3) The nonmotorized mode is more popular among the inside commuters for their shorter commute distance and lower travel time. Therefore, the public transportation and the automobile, both of which are suitable for long-distance travel, take lower shares of the total trips in the historic district.

## 5. Modeling Results

### 5.1. SEM Model Specification

The aim of this paper is to explore the influence of individual and household attributes on travel characteristics of commuters in the historic district, relating to subsistence activity, trip chain, and travel mode. Based on previous research, individual and household, participated activities are highly related to travelers' travel characteristics, and it means that individual and household attributes of travelers can not only directly affect their travel (i.e., number of trips and mode choice), but also indirectly influence it by influencing activities which they participate in.

**Figure 2:**
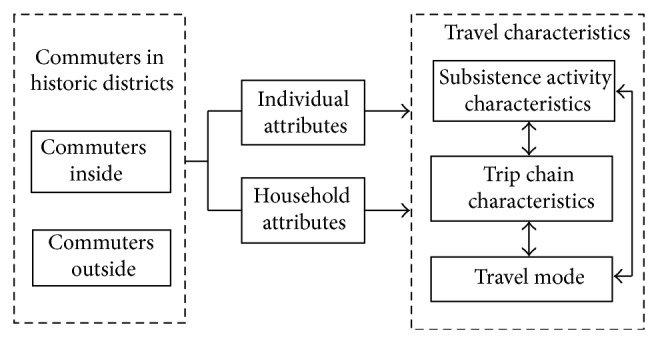
Model structure in the SEM.

In the paper, a model with 7 endogenous variables and 7 exogenous variables relating to commuters travel characteristics is established to obtain the interrelationships among these variables.  Figure 2 illustrates the initial conceptual model structure. Using the initial model framework, we developed two models for the two groups, one for commuters in the historic district, and the other for commuters out of the district. The following step is to modify the model. The hypotheses can be adjusted and the model can be retested. The model can be adjusted by adding new pathways or removing the original pathways. The final model is decided by the reported statistics.

SEM can be developed in the statistical package software named AMOS, and the estimation can be efficiently achieved by ML (maximum likelihood estimation). As the understandable result of ML, we applied it to investigate the total, direct, and indirect effects of exogenous variables on endogenous variables. Finally, we established the models for inside and outside commuters separately and discussed the estimation results, respectively.

### 5.2. Results for Inside Commuters

The estimation result for inside commuters is shown in Table 5. Also, the total effects, direct effects, and indirect effects of exogenous variables on endogenous variables are listed in the table. The goodness-fit model is provided (*χ* ^2^ = 46.77, *χ* ^2^/*df* = 2.205). The goodness of fit index (GFI) of the SEM is 0.991, which is above the recommended value 0.9, and the root mean square error of approximation (RMSEA) is 0.038 (<0.05), indicating these measures meet the acceptable criteria. The adjusted goodness of fit index (AGFI) = 0.959 is above the recommended value 0.9. All of the indices meet the criteria.

In the model for inside commuters, three exogenous variables (number of trips, commute trip numbers, and commute time) are incorporated to the individual and household characteristics. The total, direct, and indirect effects of exogenous variables on endogenous variables are shown to be consistent with the existing studies [8–10].

Regarding the variable “gender,” it has a positive influence on commute trip number. With the increasing age the commute trips on workdays will be raised. It indicates that older commuters are more likely to return home at noon, which brings an increase in trips and commute trips, and “HWHWH” and “HWOH” are the main trip chains of this group.

In terms of variable “occupation,” it poses a positive effect on travel mode. Table 5 also shows that the household annual income and ownership of automobiles act on travel mode positively, and it can be explained that occupation affects the income of commuters, and the high-income group is more likely to travel by automobile. The estimation result reveals that higher income commuters have more trips for entertainment after work, and most of them follow the “HWOH” trip chain. Nonetheless, the ownership of automobiles has a negative effect on commute time, and the reason is that the high-speed automobiles can reduce the travel time effectively.

Then it comes to the variable “gender,” and many variables relating to travel characteristics (number of trips, commute trip number, travel mode, trip chain, number of trip chains, and duration of the commuting) are influenced by it in a large degree. Usually, women play a key role in daily life and a lot of chores are left to them, resulting in large increases in number of trips and home-based trip chains. Compared with the male, it takes much longer time for their noncommuting trips, and their corresponding working time and commute time are shorter. As a result, the gender “female” has a negative effect on duration of the commuting. Similarly, gender exerts a negative influence on travel mode. It is due to that the female prefer the nonmotorized mode and public transportation.

The following variable turns to the exogenous variable “preschool children,” and it poses a positive influence on commute time, indicating that families with a preschool child will take longer time for commuting. But the household size poses a negative influence on number of trips, travel mode, and number of trip chains. With an extending household size, burdens of the family are much heavier, and family members are more likely to spend their time to share the tasks referring to maintenance activities, so the trips for other purposes and daily trips are substantially decreased. Their trip chains are featured as the simple one “HWH,” and the frequency of the chain is lessened accordingly.

As is shown above, the trips of inside commuters are mainly concerned with their work and the number of the commuting is approximate to that of trips. If the frequency of trips is increased, it will bring an increase in travel time, but the time for work will be shortened. It accords with the explanation that commute time and number of the commuting are positively correlated.

### 5.3. Results for Outside Commuters

For outside commuters, estimation of the model is shown in Table 6. The good fit to the data of the model is provided (*χ* ^2^ = 31.89, *χ* ^2^/*df* = 1.227). The goodness of fit index (GFI) of the SEM is 0.982 (>0.9), and the root mean square error of approximation (RMSEA) is 0.03 (<0.05), indicating these measures meet the acceptable criteria. The adjusted goodness of fit index (AGFI) = 0.927 is above the recommended value 0.9. These indices indicate that the final model is a good fit. Compared with the model for commuters out of the district, there are only two additional exogenous variables (number of trip chains and number of trips). The exogenous variables, gender and household size, are not significantly related to travel characteristics of outside commuters.

The occupation exerts more influences on commuters' travel characteristics, and it is positively related to the number of trips, commute trip number, mode choice, and trip chain. The coefficients indicate that compared with workers, officials and the self-employed are more inclined to return home at noon and travel for other purpose, so it results in an increase in trips and commute trips. At the same time, those people are more willing to choose a free travel mode, such as automobiles.

The gender is negatively related to mode choice, while it poses a positive influence on trip chains. They can be explained in the same way as that of inside commuters. For commuters outside of the district, most families with preschool children prefer the public transportation and the nonmotorized mode. Higher-income families are more likely to travel by automobile; then the time taken will be relatively decreased, which makes sense that the ownership of automobiles and the household income usually influence travel characteristics of commuters.

Moreover, the additional exogenous variable (number of trip chains) has a positive effect on commute time and mode choice, and the number of trips exerts a positive influence on commute time. Figure 1 shows that subsistence activity of outside commuters makes up 94.2% of the total; the trips and trip chains are increased along with increasing commute trips, so the commute time is extended as well.

## 6. Conclusions and Recommendations

Based on the household survey data in the historic district of Yangzhou, China, this study explored the relationships between the individual and household attributes and commuters' travel characteristics. First, commuters were categorized into two groups according to their working locations, which were the commuters in the historic district and the commuters out of the historic district. Then, the SEM models were estimated separately for the two commuter groups. The study analyzed the influences of individual and household attributes on the travel characteristics of different groups, which are specified by the commute time, duration of the commuting, commute trip number, number of trips on a working day, number of trip chains, the numbers of three typical home-based trip chains, and travel mode.

The comparison of the two groups showed that the commuters within historic district traveled more frequently than those outside of the district, especially in the daily trip number and trip chain times. Most commuters in the historic district have shorter trips for work, and thus they are more inclined to use nonmotorized mode. As a long commute distance for commuters out of the district, mostly they follow the trip chains named “HWH,” and they are more likely to travel by automobile. With the transition of industries in Yangzhou, more employment chances are provided in the areas out of the historic district, and more people will travel long commute trips by automobile, which will result in severe congestions on roads. Therefore, the primary thing for the inside commuters is to improve the nonmotorized travel conditions. But for the outside commuters the most needed thing is to improve the service quality of public transpiration, which is of significance for the improvement of transit usage.

The SEM was applied to analyze the influencing factors on the travel characteristics for the inside and outside commuters. The analysis results were summarized into four points: first, the age and household size have remarkable influences on the travel characteristics of the inside commuters, while they have no significant influences on that of the outside commuters. Second, in the model for inside commuters, occupation has a significant effect on the travel mode. But in the model for outside commuters, it poses significant effects on several travel characteristics, including number of trips, commute trip number, number of trip chains, and travel mode. Conversely, gender exerts significant influences on numerous travel characteristics of inside commuters, including number of trips, commute trip number, trip chains, number of trip chains, and duration of the commuting. While in the model for the outside commuters it only exerts influences on the two endogenous variables, trip chains, and travel mode. Third, preschool children has a significant influence on commute time of the inside commuters and has a certain effect on the travel mode of the outside commuters. Forth, household annual income and ownership of automobiles exert similar influences in the models for both inside and outside commuters.

The analysis is exclusively focused on travel characteristics of commuters living in the historic center of Yangzhou, while the travel characteristics of commuters living out of the district still have not been incorporated in the study. As a secondary city of Yangzhou, its historic district is the center of politics, economy, and culture. Most residents in this area are commuters. But, in large cities such as Nanjing, the working places of residents in historic district are usually located in the outside, which is quite different from that of Yangzhou. So the differences of their travel behavior remained to be an important topic to be studied in the future.

## Figures and Tables

**Table 1:** Description of exogenous variables in SEM.

Attributes	Exogenous variable	Description
Household attributes	Household size (HS)	HS = 1: 1 or 2 persons; HS = 2: 3 persons; HS = 3: more than 3 persons
	Preschool children (CHI)	CHI = 1: yes; CHI = 0: no
	Automobile ownership (AUTO)	AUTO = 1: yes; AUTO = 0: no
	Annual household income (INC)	INC = 1: <30000; INC = 2: 30000–50000; INC = 3: >50000

Individual attributes	Gender (GEN)	GEN = 1: male; GEN = 2: female
	Occupation (OCC)	OOC = 1: worker or attendant; OCC = 2: staff or official; OCC = 3: self-employed
	Age (AGE)	AGE = 1: <30 years old; AGE = 2: 30–50 years old; AGE = 3: >50 years old

**Table 2:** Description of endogenous variables in SEM.

Category	Variables	Description
Subsistence activity	Commute time	Time allocated to work or work-related trips
	Commute trip number	Number of work or work-related trips
	Duration of the commuting	Successive time of work or work-related activities in the workday

Travel behavior	Number of trips	The total number of trips in a whole day
	Number of trip chains	Number of home-based trip chains
	Type of trip chain	A sequence of trips that starts and ends at the household location in a whole day

Travel mode	Mode choice	Nonmotorized mode; public transportation; automobile

**Table 3:** Descriptions of characteristics of inside and outside commuters.

Variables	Description	Inside commuters (*N* = 705)		Outside commuters (*N* = 245)	
		Sample size	Percent (%)	Sample size	Percent (%)
Household characteristics					
Household size	1 or 2 persons	120	17	40	16.3
	3 persons	412	58.4	140	57.1
	More than 3 persons	173	24.5	65	26.5
Preschool children	Yes	164	23.3	34	13.9
	No	541	76.7	211	86.1
Automobile ownership	Yes	345	48.9	129	52.7
	No	360	51.1	116	47.3
Annual household income (CNY)	<30000	415	58.9	130	53.1
	30000–50000	197	27.9	80	32.7
	>50000	93	13.2	35	14.3
Individual characteristics					
Gender	Male	391	55.5	151	61.6
	Female	314	44.5	94	38.4
Occupation	Worker or attendant	449	63.7	183	74.7
	Staff or official	179	25.4	38	15.5
	Self-employed	77	10.9	24	9.8
Age	<30 years old	180	25.5	58	23.7
	30–50 years old	425	60.3	157	64.1
	>50 years old	100	14.2	30	12.2

**Table 4:** Travel characteristics of inside and outside commuters.

Travel characteristic	Commuters in the district	Commuters out of the district
	Mean (*N* = 705)	Mean (*N* = 245)
Subsistence activity		
Duration of the commuting	508.68 min	539.7 min
Commute time	51.16 min	62.2 min
Commute trip number	2.7 times per day	2.26 times per day
Trip chain		
Number of trips	2.88 times per day	2.4 times per day
Number of trip chains	1.41	1.17
HWH	58.40%	82%
HWHWH	29.20%	8.60%
HWOH	12.30%	9.40%
Travel mode		
Nonmotorized mode	69.10%	56.70%
Public transportation	4%	8.20%
Automobile	27%	35.10%

**Table 5:** Effects among exogenous and endogenous variables of commuters in the historic district.

Variable	Effect	Age	Occupation	Gender	Preschool children	Household annual income	Automobile	Household size	Number of trips	Commute trip number	Commute time
Number of trips	Overall effect	0.106	—	0.267	—	—	—	−0.168	—	—	—
	Direct effect	0.106	—	0.267	—	—	—	−0.168	—	—	—
	Indirect effect	0	—	0	—	—	—	0	—	—	—

Commute trip number	Overall effect	0.081	—	0.025^*^	—	—	—	—	0.38^*^	—	—
	Direct effect	0.041	—	−0.077^*^	—	—	—	—	0.38^*^	—	—
	Indirect effect	0.04	—	0.102^*^	—	—	—	—	0	—	—

Commute time	Overall effect	—	—	—	4.43	—	−3.612	—	—	19.456^*^	—
	Direct effect	—	—	—	4.208	—	−3.581	—	—	19.456^*^	—
	Indirect effect	—	—	—	0.222	—	−0.031	—	—	0	—

Mode choice	Overall effect	—	0.102^*^	−0.543^*^	—	0.111	0.833^*^	−0.14^*^	—	—	−0.002
	Direct effect	—	0.098^*^	−0.544^*^	—	0.11	0.826^*^	−0.141^*^	—	—	−0.002
	Indirect effect	—	0.004^*^	−0.001^*^	—	0.001	0.007^*^	−0.001^*^	—	—	0

Trip chain	Overall effect	—	—	0.249^*^	—	0.026	—	−0.114	—	—	—
	Direct effect	—	—	0.249^*^	—	0.026	—	−0.114	—	—	—
	Indirect effect	—	—	0	—	0	—	0	—	—	—

Number of trip chains	Overall effect	—	—	0.134^*^	—	—	—	−0.069	—	—	—
	Direct effect	—	—	0.134^*^	—	—	—	−0.069	—	—	—
	Indirect effect	—	—	0	—	—	—	0	—	—	—

Duration of the commuting	Overall effect	—	—	−45.868^*^	—	—	—	—	−49.15^*^	—	—
	Direct effect	—	—	−32.755^*^	—	—	—	—	−49.15^*^	—	—
	Indirect effect	—	—	−13.113^*^	—	—	—	—	0	—	—

Notes: *N* = 705; *χ* ^2^ = 46.577, df = 23, *χ* ^2^/df = 2.205; GFI = 0.991; AGFI = 0.959; RMSEA = 0.038.

“—” indicates no significant influence; “∗” indicates *P* ≤ 0.001; other values indicate *P* ≤ 0.05.

**Table 6:** Direct and indirect effects between exogenous and endogenous variables of SEM for outside commuters.

Variable	Effect	Age	Occupation	Gender	Preschool children	Household annual income	Automobile	Household size	Number of trip chains	Number of trips
Number of trips	Overall effect	—	0.124	—	—	—	—	—	—	—
	Direct effect	—	0.124	—	—	—	—	—	—	—
	Indirect effect	—	0	—	—	—	—	—	—	—

Commute trip number	Overall effect	—	0.055	—	—	—	0.091	—	—	—
	Direct effect	—	0.039	—	—	—	0.086	—	—	—
	Indirect effect	—	0.016	—	—	—	0.005	—	—	—

Commute time	Overall effect	—	—	—	—	−4.809	—	—	11.691^*^	44.507^*^
	Direct effect	—	—	—	—	−4.554	—	—	11.691^*^	44.507^*^
	Indirect effect	—	—	—	—	−0.255	—	—	0	0

Mode choice	Overall effect	—	0.121	−0.504^*^	−0.429	0.215	0.878^*^	—	0.55	—
	Direct effect	—	0.121	−0.504^*^	−0.429	0.215	0.878^*^	—	0.55	—
	Indirect effect	—	0	0	0	0	0	—	0	—

Trip chain	Overall effect	—	0.087	0.155	—	—	—	—	—	—
	Direct effect	—	0.087	0.155	—	—	—	—	—	—
	Indirect effect	—	0	0	—	—	—	—	—	—

Number of trip chains	Overall effect	—	—	—	—	—	—	—	—	—
	Direct effect	—	—	—	—	—	—	—	—	—
	Indirect effect	—	—	—	—	—	—	—	—	—

Duration of the commuting	Overall effect	—	—	—	—	—	—	—	—	—
	Direct effect	—	—	—	—	—	—	—	—	—
	Indirect effect	—	—	—	—	—	—	—	—	—

Notes: *N* = 245; *χ* ^2^ = 31.89, df = 26, *χ* ^2^/df = 1.227; GFI = 0.982; AGFI = 0.927; RMSEA = 0.03.

“—” indicates no significant influence; “∗” indicates *P* ≤ 0.001; other values indicate *P* ≤ 0.05.
